# University rankings and medical physics

**DOI:** 10.1007/s13246-013-0234-9

**Published:** 2013-12-07

**Authors:** Clive Baldock

**Affiliations:** Executive Dean of Science, Macquarie University, North Ryde, NSW 2109 Australia

## Introduction

In recent years there has been a move to quantify many aspects of higher education productivity. To this end, this author previously published on the role of the h-index and how it is being used as a quantifier of research productivity of the individual researcher [1, 2].

Also of interest in recent years has been the focus in higher education circles with regards to league tables and the rankings of universities, with much anticipation each year among university administrators, funding agencies and students when the various ranking agencies publish their latest ranking lists [3]. Such rankings, now a standard feature, are playing a significant role in a changing higher education landscape with implications for many.

Despite ongoing debates about the use and validity of university rankings, they are becoming increasingly popular and enabling students as consumers to compare institutions within a country and around the world as they make decisions regarding which university to potentially attend. Further, many university vice-chancellors, presidents, rectors and administrators see rankings as having potential influence on their organisational missions, strategies, personnel, recruitment, and public relations [4, 5]. Furthermore, rankings often drive the allocation of resources with decision makers and administrators sensitive to the resulting prestige that may be associated with ranking performance [6]. Government and funding agencies are also increasingly using rankings as a policy instrument to assess the performance of higher education institutions [7].

As well as university rankings and league tables occupying the attention of higher education leaders and policy makers, much has been written in the literature on this topic along with numerous international conferences and seminars having been held [8].

## History of university rankings

The practice of university rankings dates back to the beginning of the twentieth century with the publication in the United Kingdom of *Where We Get Our Best Men*. In this study the backgrounds of “England’s most prominent and successful men of the time” were evaluated with particular reference to where each studied. A consequence was the listing of universities ranked by the number of distinguished alumni that the ranked universities could lay claim to [9].

Subsequently in 1925, graduate programs in United States universities were ranked on the basis of peer reputation [10]. Significant published rankings of universities however did not commence until 1983 when the US News and World Report started ranking college undergraduate education programs with this ranking becoming an annual event from 1987.

Since 2003 numerous university rankings have been published with some now becoming particularly well known and popular. Some of the most well known rankings include the academic ranking of world universities (ARWU) from Shanghai Jiao Tong University in China, the QS World University Rankings, the Times Higher Education World University Rankings and more recently the Leiden University Rankings.

By 2009 at least 33 ranking systems of higher education systems from around the world were in use [11] and by 2011 at least 50 national ranking systems and 10 ranking systems of global significance were being published [8].

## How rankings and league tables work

League tables and university rankings endeavor to simplify and summarise entire institutions into single, numerical comparators or indicators. Rankings systems operate by comparing institutions on a range of comparators, with the number of comparators varying significantly, from just a few in the simplest case to several dozen in the case of the most complicated. Specific areas of institutional activity or types of institutional output can therefore be compared between institutions [12].

In most cases, league table systems use comparator data to calculate a composite score such as a university’s research publication output and its reputation. Once scores have been derived for each comparator, they are generally weighted according to importance. The weighted scores from all comparators are then summed to calculate an overall final score for each institution. The choice of comparator and the weight given to each makes an enormous amount of difference in the final output with the publishers of the rankings generally deciding the choice of comparators and weightings and in so doing defining so-called ‘quality’.

Many have long criticized what they describe as the inflated influence of university rankings, saying that their methodology and data are problematic [13]. Many critics have pointed out that the methodologies used tend to focus too much on research, and pay insufficient attention to other key factors, such as other forms of scholarship to that of research and how well a university teaches its students to think critically and to innovate [3].

## Examples of university rankings and the ranking of Australian universities

The three most influential and widely observed international university rankings are the Academic Ranking of World Universities (ARWU), the QS World University Rankings and the Times Higher Education World University Rankings.

### Academic Ranking of World Universities (ARMU)

ARWU is compiled by the Shanghai Jiao Tong University in China and has provided an annual global ranking of universities since 2003. It was originally funded by the Chinese government to measure the gap between Chinese and so-called ‘world class’ universities. As a comparator, ARWU includes the number of articles published by Nature or Science and the number of nobel prize winners and fields medalists (mathematics). A criticism of the ARWU ranking is that it is biased towards research and the sciences and does not measure the quality of teaching. Table 1 shows the 2013 ARWU Ranking of Australian universities.

**Table 1:** 2013 ARWU ranking of Australian universities

University	Australian rank	World rank
University of Melbourne	1	54
Australian National University	2	66
University of Queensland	3	85
University of Western Australia	4	91
University of Sydney	5	97
Monash University	6–7	101–150
University of New South Wales	6–7	101–150
Macquarie University	8–9	201–300
University of Adelaide	8–9	201–300
Flinders University	10–16	301–400
Griffith University	10–16	301–400
James Cook University	10–16	301–400
Swinburne University of Technology	10–16	301–400
University of Newcastle	10–16	301–400
University of Tasmania	10–16	301–400
University of Wollongong	10–16	301–400
Curtin University	17–19	401–500
La Trobe University	17–19	401–500
University of Technology Sydney	17–19	401–500

http://www.australianuniversities.com.au/rankings. Accessed 23 Nov 2013.

### QS World University Rankings

The QS World University Rankings are annual university rankings published by QS and provides an overall rankings as well as ranking for individual subjects. QS originally published its rankings with the times higher education from 2004 to 2009 as the times higher education-QS world university rankings. Their collaboration however ended in 2010. QS subsequently published solely using the pre-existing methodology, while times higher education created a new ranking with Thomson Reuters, published as the times higher education world university rankings. Table 2 shows the 2013 QS World University Ranking of Australian universities.

**Table 2:** 2013 QS World University Ranking of Australian Universities

University	Australian rank	World rank
Australian National University	1	27
University of Melbourne	2	31
University of Sydney	3	38
University of Queensland	4	43
University of New South Wales	5	52
Monash University	6	69
University of Western Australia	7	84
University of Adelaide	8	104
Macquarie University	9	263
University of Technology Sydney	10	272
University of Wollongong	11	276
Queensland University of Technology	12	279
Curtin University	13	284
RMIT University	14	291
University of Newcastle	15	298
Griffith University	16	341
University of South Australia	16	341
James Cook University	18	351
Deakin University	19	380
La Trobe University	20	390
University of Tasmania	21	401–410
Bond University	22	421–430
Flinders University	23	431–440
Charles Darwin University	24	471–480
Swinburne University of Technology	25	481–490
Murdoch University	26	551–600
University of Canberra	27	601–650
University of Western Sydney	28	651–700
University of New England	29	701+
University of Southern Queensland	29	701+
Victoria University	29	701+

http://www.australianuniversities.com.au/rankings. Accessed 23 Nov 2013.

### The Times Higher Education World University Rankings

The Times Higher Education World University Rankings (or THE World University Rankings) are annual world university rankings published by the Times Higher Education (THE) with data supplied by Thomson Reuters that provides citation database information. Included are overall and the subject rankings. Table 3 shows the 2013 Times Higher Education World University Ranking of Australian universities.

**Table 3:** 2013 Times higher education world university ranking of Australian universities

University	Australian rank	World rank
University of Melbourne	1	34
Australian National University	2	48
University of Queensland	3	63
University of Sydney	4	72
Monash University	5	91
University of New South Wales	6	114
University of Western Australia	7	168
University of Adelaide	8	201–225
University of Newcastle	9	251–275
Macquarie University	10	276–300
Queensland University of Technology	10	276–300
University of Wollongong	10	276–300
Deakin University	13	301–350
Murdoch University	13	301–350
University of South Australia	13	301–350
University of Technology Sydney	13	301–350
Charles Darwin University	17	351–400
Swinburne University of Technology	17	351–400
University of Tasmania	17	351–400

http://www.australianuniversities.com.au/rankings. Accessed 23 Nov 2013.

## Relevance to medical physics

As indicated in this article, university rankings enable students as consumers to compare institutions within a country and around the world as they make decisions regarding which university to attend. Students will potentially make future choices of what and where to study, whether it be a postgraduate course in medical physics or enrolling in a PhD in biomedical engineering, and based on where a university lies in a particular ranking. Such choices will not necessarily be based on which postgraduate academic programs are of higher ‘quality’.

To those working in a clinical environment in the disciplines of medical physics or biomedical engineering, such issues that face those in universities are not always evident. To this end, this article brings the issue of league tables and university rankings to the attention of individuals. This is of particular importance as more practicing medical physicists and biomedical engineers aspire to have an academic aspect to their portfolio of activities.
